# Anti-glycative effects of asiatic acid in human keratinocyte cells

**DOI:** 10.7603/s40681-014-0019-9

**Published:** 2014-08-13

**Authors:** Zhi-Hong Wang

**Affiliations:** Department of Medical Research, China Medical University Hospital, Taichung, Taiwan

**Keywords:** HaCaT cell, Glycation, Asiatic acid, RAGE, MAPK

## Abstract

Background: Human skin keratinocyte (HaCaT) cells served to examine effects of asiatic acid (AA) at 1, 2, 4 and 8 μM against advanced glycative endproduct (AGE)-modified bovine serum albumin (BSA) induced glycative stress.

Results: AGE-BSA treatment reduced cell viability; and increased reactive oxygen species, nitric oxide, protein carbonyl, interleukin (IL)-1beta and tumor necrosis factor-alpha levels in HaCaT cells. Yet AA pretreatments decreased these oxidative and inflammatory factors, dose-dependently lowering nitric oxide synthase activity and expression. AGE-BSA raised activity and expression of caspase-3 and caspase-8. AA pretreatments at 2-8 μM decreased activity and expression of these two caspases. AGE-BSA declined collagen I expression, but enhanced matrix metalloproteinase (MMP)-1, MMP-8 and MMP-9 protein expression. AA pretreatments at 2-8 μM maintained collagen I expression, and reduced three MMPs expression. AGE-BSA also up-regulated RAGE (receptor of AGE), p-p38 and p-JNK expression. AA pretreatments at 2-8 μM suppressed RAGE expression, and at 1-8 μM down-regulated p-p38 and p-JNK expression.

Conclusion: Asiatic acid, via its anti-glycative activity, could protect skin. Thus, this compound could be developed as an external agent and applied for personalized medicine.

## 1. Introduction

Glycation emanating from ultraviolet light, genetic factors and environmental pollution promotes skin aging [1,2]. Glycation causes advanced glycative endproducts (AGEs) to accumulate in skin, especially in long-lived proteins such as dermal elastin and collagen during skin aging [3,4]. AGE, by reacting with its receptor, RAGE, could activate mitogen-activated protein kinase (MAPK) pathway to promote oxidative and inflammatory reactions in skin, which results in overproduction of free radicals like nitric oxide (NO) and reactive oxygen species (ROS), as well as inflammatory cytokines like interleukin (IL)-6 and tumor necrosis factor (TNF)-alpha [5,6]. Both oxidative and inflammatory stress can enhance skin cell aging, and even apoptosis. Molinari et al. [7] and Zhu et al. [8] reported that AGEs could induce expression of matrix metalloproteinases-9 (MMP-9) and MMP-8 in normal human dermal fibroblasts and keratinocyte (HaCaT) cells. Excessive expression of MMPs promotes skin aging by destabilizing extracellular matrix (ECM), enhancing lysis of dermal collagen and elastin fibers [9,10]. Increased expression of MMP-1, major MMP responsible for degradation of Type I collagen, favors skin aging, with collagen I as most abundant structural protein in skin connective tissue [11]. Thus, any agent with ability to impede AGE-RAGE interaction, suppress MAPK pathway, reduce MMP expression, or attenuate oxidative and inflammatory stress may provide anti-glycative protection and benefit skin cell survival.

**Fig. 1:**
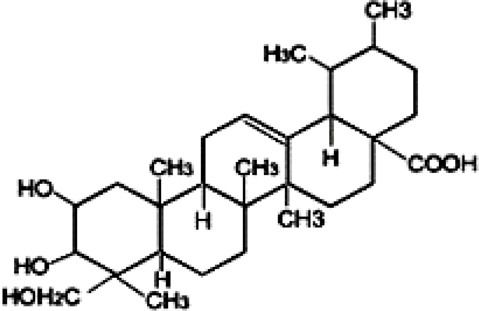
Structure of asiatic acid.

Asiatic acid, a pentacyclic triterpene (Figure 1), naturally occurs in many vegetables and fruits: e.g., glossy privet fruit (*Ligustrum lucidum* Ait.), basil (*Ocimum basilicum*), brown mustard (*Brassica juncea*) [12]. This triterpene reportedly has anti-oxidative and anti-inflammatory properties [12,13]. The protection by asiatic acid at 5 μM against ultraviolet-A induced ROS and MMPs formation in HaCaT cells has been cited [14,15]. Masoko et al. [16] revealed that asiatic acid benefitted wound healing via anti-fungal activity. Bonte et al. [17] indicated this triterpene stimulating synthesis of Type I collagen. Its inhibitory effects upon NO production and inducible nitric oxide synthase (iNOS) expression in mice skin tumor or skin cells have been observed [18,19]. Prior studies suggest that asiatic acid seems an anti-aging agent for skin, yet it remains known whether this triterpene can protect skin cells against glycation-associated injury. In addition, asiatic acid can regulate MAPK in RAW 264.7 macrophage cells [20] and protect neurons against ceramide-induced apoptosis via downregulating caspase-3 [21]. So far, little attention has been paid to its impact on RAGE, MAPK and caspase pathways in skin cells. In our study, AGE-modified bovine serum albumin (BSA) induced glycative stress in HaCaT cells to rate effect of asiatic acid at various doses against AGE-BSA-induced, glycationassociated oxidative and inflammatory injury to HaCaT cells. Influence of this triterpene on activity and/or protein expression of caspases, MMPs, RAGE and MAPK were also evaluated.

## 2. Materials and methods

### 2.1. Chemicals

Asiatic acid (AA, 98%) was obtained from Sigma-Aldrich Co. (St. Louis, MO); AGE-BSA from Calbiochem Co. (San Diego, CA); medium, plates, antibiotics, and chemicals used for cell culture from Difco Laboratory (Detroit, MI). Chemicals used were of the highest purity commercially available.

### 2.2. Cell culture

Human skin keratinocyte (HaCaT) cell line from American Type Culture Collection (ATCC, Rockville, MD) were cultured in Dulbecco’s modified Eagle’s medium (DMEM) supplemented with 10% fetal bovine serum, 100 U/mL penicillin and 100 U/mL streptomycin under 95% air/ 5% CO_2_ at 37°C. Culture medium was changed every three days, cells subcultured once a week, medium changed to serum-deprived medium, cells washed with serum-free DMEM 24 hr before experiments and replanted in 96-well plates. Phosphate buffer saline (PBS, pH 7.2) was used to adjust cell number to 10^5^/ml for various experiments and analyses.

### 2.3. Experimental design

Cytotoxic effects of AGE-BSA at 25, 50, 100, 200 and 400 μg/ml upon HaCaT cells were examined. Results showed AGE-BSA at these doses yielded 91, 73, 49, 24, and 8% cell viability compared to controls in which AGE-BSA was not added. Thus, AGE-BSA at 100 μg/ml, led to about 50% cell death, further examined anti-glycative effects of asiatic acid. AA was dissolved in dimethyl sulfoxide (DMSO), then diluted with medium. Final DMSO concentration of in medium was 0.5%, at which DMSO never affected measurements (data not shown). HaCaT cells (10^5^ cells/ml) were treated with AA at 1, 2, 4, or 8 μM for 36 hr at 37°C, which led to 95-97% incorporation of test agent into cells. After washing with serum-free DMEM, cells were exposed to AGE-BSA at 100 μg/ml for 24 hr. Control groups were HaCaT cells containing neither AGE-BSA nor AA.

### 2.4. Cell viability

3-(4,5-Dimethylthiazol-2-yl)-2,5-Diphenyltetrazolium bromide (MTT) assay examined cell viability. Briefly, HaCaT cells were incubated with 0.25 mg MTT/ml for 3 hr at 37°C. Amount of MTT formazan product was derived by absorbance at 570 nm (630 nm as reference), using a microplate reader (Bio-Rad, Hercules, CA); viability was expressed as percentage of control groups.

### 2.5. Glutathione (GSH), ROS, protein carbonyl, and glutathione peroxide (GPX) activity

Cells washed twice with PBS (pH 7.2) were scraped from plates and homogenized in PBS containing 0.5 mM butylated hydroxytoluene to prevent sample further oxidation. The homogenate was centrifuged at 3000 xg for 20 min at 4°C, supernatant used for assays, as per manufacturer’s instructions. GSH concentration (ng/mg protein) was quantified by commercial colorimetric assay kit (OxisResearch, Portland, OR), ROS level determined by oxidation sensitive dye, 2',7'-dichlorofluorescein diacetate, cells incubated with 50 μM dye for 30 min and washed with PBS. Fluorescence was evaluated by microplate reader at excitation and emission wavelengths of 485 and 530 nm, respectively; relative fluorescence unit (RFU) was the difference in fluorescence values obtained at time 0 and 5 min. Results are expressed as RFU/mg protein. Protein carbonyl level was determined with the Zentech PC kit (BioCell, Auckland, New Zealand). Activity (U/mg protein) of GPX was determined by assay kit (EMD Biosciences, San Diego, CA), protein concentration measured by Bio-Rad protein assay reagent (Bio-Rad Laboratories Inc. Hercules, CA).

### 2.6. Assay for NO and NOS activity

NO yield was measured by formation of nitrite. Briefly, 100 μL supernatant treated with nitrate reductase, NADPH, and FAD was incubated for 1 hr at 37°C in the dark. After centrifuge at 6,000 xg, supernatant was mixed with Griess reagent for color development. Absorbance at 540 nm was compared with sodium nitrite standard curve, method described in Sutherland et al. [22] used to gauge total NOS activity by incubating 30 μL homogenate with 10 mM NADP, 10 mM L-valine, 3000 U/ml calmodulin, 5 mM tetrahydrobiopterin, 10 mM CaCl_2_, and 100 μM L-arginine mixture containing L-[^3^H]arginine.

### 2.7. Measurement of caspase activity

Activity of caspase-3 and -8 was detected by using fluorometric assay kits (Upstate, Lake Placid, NY) according to the manufacturer’s protocol. The intra-assay CV was 3.1-4.3%, inter-assay CV 5.0-5.9%. In brief, control or treated cells were lysed and incubated in ice for 10 min. Cell lysate (50 μl) was mixed with 50 ml of reaction buffer and 5 ml of fluorogenic substrates specific to caspase-3 or -8 in a 96-well microplate. After incubation at 37°C for 1 hr, fluorescent activity was measured by fluorophotometer with excitation at 400 nm and emission at 505 nm. Data were expressed as a percentage of control groups.

### 2.8. Western blot analysis

HaCaT cells homogenized in buffer containing 0.5% Triton X-100 and protease-inhibitor cocktail (1:1000, Sigma-Aldrich Chemical Co., St. Louis, MO) were further mixed with buffer (60 mM Tris-HCl, 2% SDS and 2% β-mercaptoethanol, pH 7.2), and boiled for 5 min. Sample at 40 μg protein was applied to 10% SDS-polyacrylamide gel electrophoresis, then transferred to nitrocellulose membrane (Millipore, Bedford, MA) for 1 hr. After blocking with solution containing 5% nonfat milk for 1 hr to prevent non-specific binding of antibody, membrane was incubated with mouse anti-GPX, anti-iNOS (1:1000), anti-caspase-3, anti-caspase-8 (1:500), anti-collagen I (1:1000), anti-MMP-1, anti-MMP-9, anti-MMP-8 (1:500), anti-RAGE and anti-MAPK (1:2000) monoclonal antibodies (Boehringer-Mannheim, Indianapolis, IN) at 4°C overnight, then reacted with horseradish peroxidase-conjugated antibody for 3.5 hr at room temperature. Detected bands were quantified by image analyzer (ATTO, Tokyo, Japan), and glyceraldehyde-3-phosphate dehydrogenase (GAPDH) served as loading control. Blot was quantified by densitometric analysis, results normalized to GAPDH and given as arbitrary units (AU).

### 2.9. Statistical analysis

Each treatment was analyzed from ten different preparations (n=10), data reported as mean ± standard deviation (SD) and subjected to analysis of variance. Differences among means were derived by Least Significance Difference Test, significance defined as *P*<0.05.

**Fig. 2:**
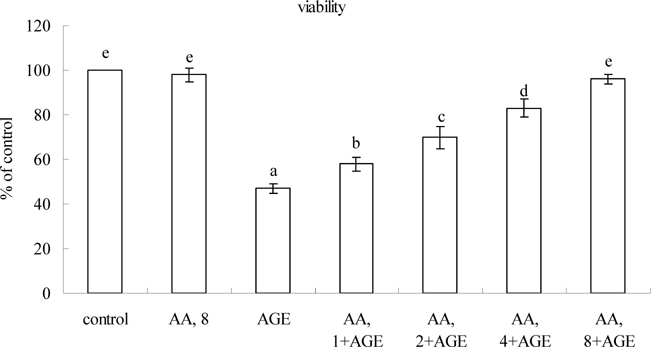
Effects of AA on viability determined by MTT assay in HaCaT cells pretreated by AA at 1, 2, 4 or 8 μM for 36 hr, followed by exposure to 100 μg/ml AGE-BSA (AGE) for 24 hr. Cells containing neither AA nor AGE-BSA as control. Data mean±SD (n=10). ^a-e^Means among bars without common letter differ, *P*<0.05.

**Table 1:** Effects of AA upon level of GSH, ROS, NO and protein carbonyl in HaCaT cells were pretreated by AA at 1, 2, 4 or 8 μM for 36 hr, followed by exposure to 100 μg/ml AGE-BSA (AGE) for 24 hr. Cells containing neither AA nor A GE-BSA as control. Data mean ±SD (n=10).

	GSH ng/mg protein	ROS RFU/mg protein	NO mmol/mg protein	Protein carbonyl pmol/mg protein
Control	92±3^e^	0.18±0.06^a^	2.1±0.5^a^	9.1±1.1^a^
AA, 8	93±4^e^	0.17±0.04^a^	2.0±0.4^a^	8.8±1.3^a^
AGE	43±2^a^	1.77±0.21^f^	17.9±1.4^f^	167.2±12.8^f^
AA, 1+AGE	53±5^b^	1.45±0.17^e^	14.8±1.1^e^	141.5±10.9^e^
AA, 2+AGE	66±3^c^	1.08±0.10^d^	11.0±1.0^d^	119.8±9.5^d^
AA, 4+AGE	82±4^d^	0.72±0.07^c^	7.4±0.7^c^	87.6±5.3^c^
AA, 8+AGE	86±5^d^	0.49±0.05^b^	4.2±0.6^b^	46.3±6.0^b^

## 3. Results

**Fig. 3:**
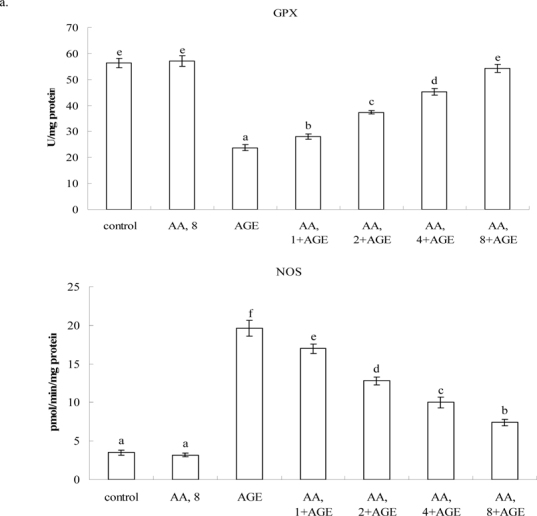

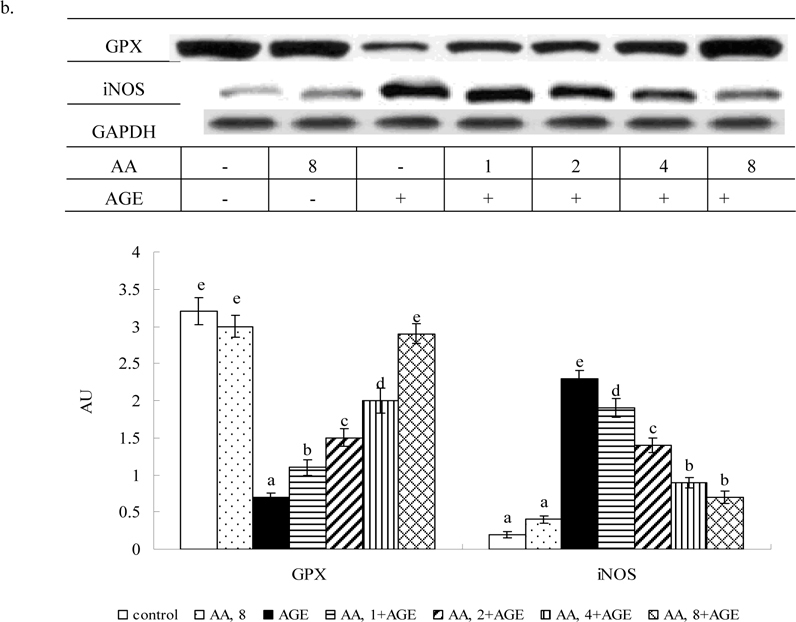
Effects of AA on activity of (a) GPX and NOS, and protein expression (b) GPX and iNOS in HaCaT cells pretreated by AA at 1, 2, 4 or 8 μM for 36 hr, followed by exposure to 100 μg/ml AGE-BSA for 24 hr. Cells containing neither AA n orAGE-BSA (AGE) as control. Data mean±SD (n=10). ^a-f^Means among bars without common letter differ, *P*<0.05.

Treatments of 8 μM AA alone in HaCaT cells (without AGE-BSA exposure) did not affect any measurement when compared to controls (*P*>0.05). Figure 2 plots AGE-BSA exposure reducing 50% viability; AA pretreatments dose-dependently enhanced viability (*P*<0.05). AGE-BSA exposure lowered GSH content 52%, raising ROS, NO and protein carbonyl levels 8.4, 8.5, and 18.3 fold, respectively (Table 1, *P*<0.05). Pretreatment with AA retained 21-85% GSH content, lowering ROS, NO, and protein carbonyl 18-72%, 17-76%, and 15-70%, respectively; dose-dependent manner was evident in decreasing ROS, NO and protein carbonyl levels (*P*<0.05). AGE-BSA lowered GPX activity 54% and protein expression 61%, yet enhanced NOS activity 5.2 fold and iNOS expression 5.5 fold (Figure 3). AA pretreatments dose-dependently retained GPX activity and expression (*P*<0.05). AA at 8 μM meant GPX activity and protein expression similar to that of controls (*P*>0.05). Pretreatments also decreased NOS activity 13-55% and iNOS expression 19-68% (*P*<0.05). AGE-BSA increased IL-1beta 6.4 fold, IL-67.3 fold, and TNF-alpha release6.1 fold in HaCaT cells (Table 2, *P*<0.05). AA pretreatments at 2-8 μM lowered IL-1beta, IL-6, and TNF-alpha formation (*P*<0.05).

**Fig. 4:**
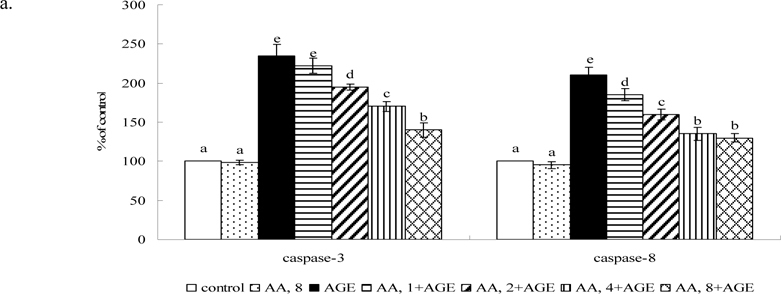

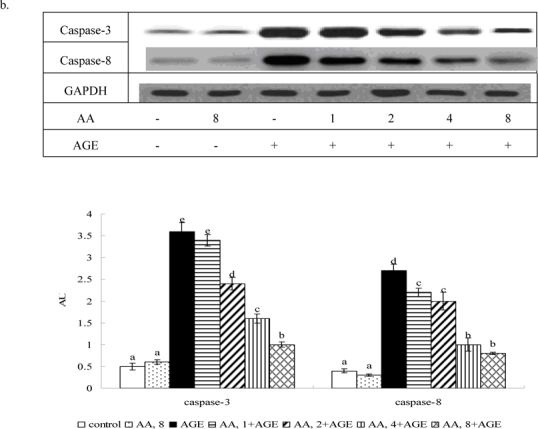
Effects of AA upon activity (a) and protein expression (b) of caspase-3 and caspase-8 in HaCaT cells pretreated by AA at 1, 2, 4 or 8 μM for 36 hr, followed by exposure to 100 μg/ml AGE-BSA (AGE) for 24 hr. Cells containing neither AA nor AGE-BSA as control. Data mean±SD (n=10). ^a-e^Means among bars without common letter differ, *P*<0.05.

**Fig. 5:**
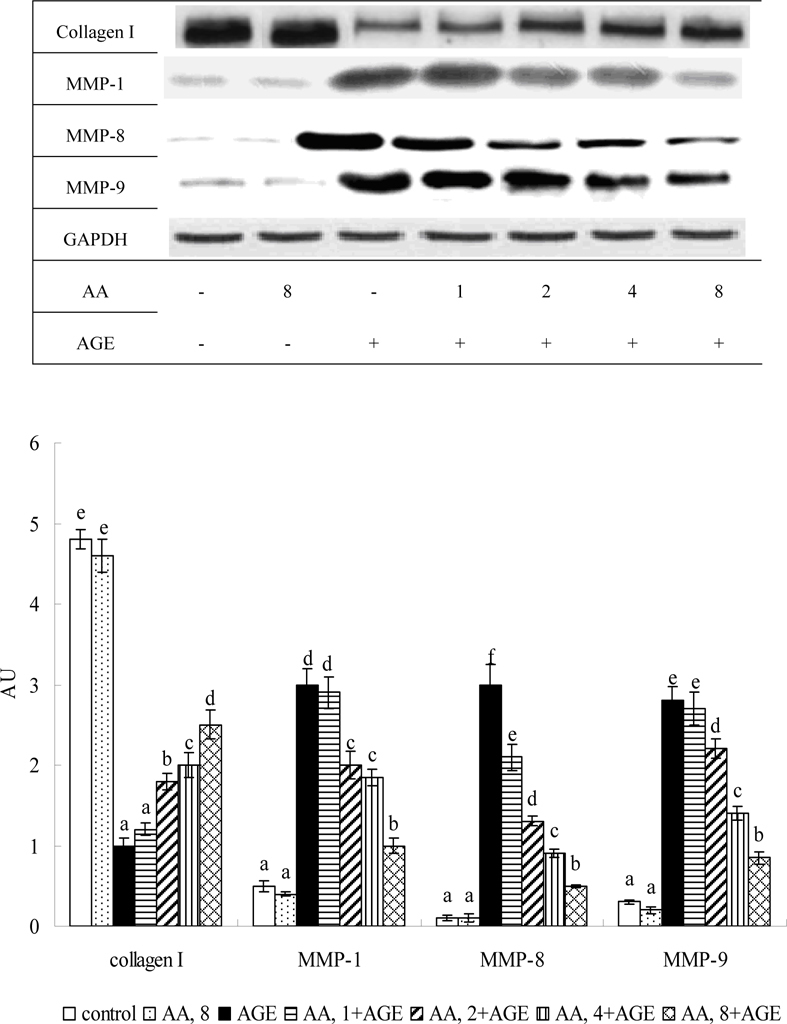
Effects of AA on protein expression of collagen I, MMP-1, MMP-8 and MMP-9 in HaCaT cells pretreated by AA at 1, 2, 4 or 8 μM for 36 hr, followed by exposure to 100 μg/ml AGE-BSA (AGE) for 24 hr. Cells containing neither AA nor AGE-BSA as control. Data mean ±SD (n=10). ^a-f^Means among bars without common letter differ, *P*<0.05.

Figure 4 shows AGE-BSA elevating caspase-3 and -8 activity 2.5 and 2.2 fold; caspase-3 and -8 protein expression 7.9 and 6.7 fold, respectively (*P*<0.05). AA pretreatment at 2-8 μM decreased caspase-3 activity and expression 18-47% and 30-58%, respectively. AA at 1-8 μM reduced caspase-8 activity and expression at 15-46% and 22-56%, respectively (*P*<0.05). AGE-BSA reduced 68% collagen I expression, but raised MMP-1, MMP-8 and MMP-9 protein expression 4.5, 3.1, and 2.7 fold (Figure 5, *P*<0.05), respectively. At 2-8 μM it maintained collagen I expression, reducing MMP-1 and -9 expression 33-65% and 17-63% (*P*<0.05), respectively, while dose-dependently lowering MMP-8 level (*P*<0.05).

**Table 2:** Effects of AA on level (pg/ml) of IL-1beta, IL-6 and TNF-alpha in HaCaT cells pretreated by AA at 1, 2, 4 or 8 μM for 36 hr, followed by exposure to 100 μg/ml AGE-BSA (AGE) for 24 hr. Cells containing neither AA nor AGE-BSA as control. Data mean ±SD (n=10).

	IL-1beta	IL-6	TNF-alpha
Control	13±2^a^	12±3^a^	16±5^a^
AA, 8	11±4^a^	9±2^a^	13±4^a^
AGE	86±6^e^	80±5^e^	98±7^e^
AA, 1+AGE	79±5^e^	72±3^e^	94±5^e^
AA, 2+AGE	67±5^d^	59±4^d^	79±3^d^
AA, 4+AGE	51±4^c^	46±3^c^	62±4^c^
AA, 8+AGE	35±3^b^	32±4^b^	48±3^b^

^a-e^Means in column without common letter differ, *P*<0.05.

**Fig. 6:**
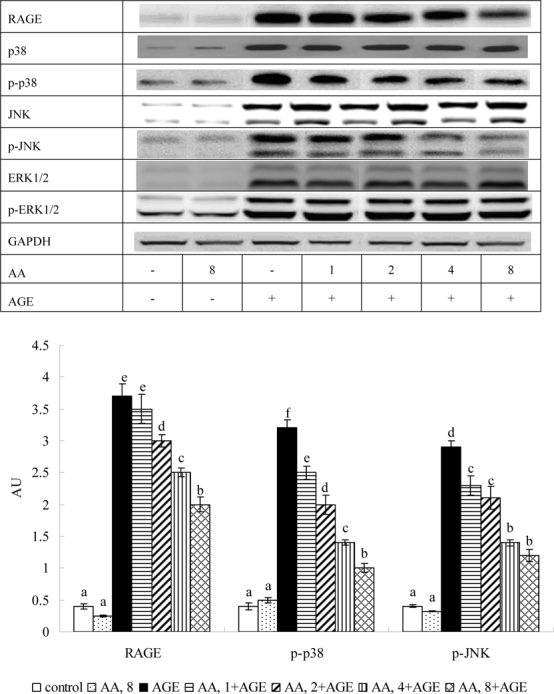
Effects of AA on protein expression of RAGE and MAPK in HaCaT cells pretreated by AA at 1, 2, 4 or 8 μM for 36 hr, followed by exposure to 100 μg/ml AGE-BSA (AGE) for 24 hr. Cells containing neither AA nor AGE-BSA as control. Data mean±SD (n=10). ^a-f^ Means among bars without a common letter differ, P<0.05.

Figure 6 shows AGE-BSA raising RAGE, p-p38 and p-JNK expression 8.8, 6.8, and 8.1 fold, respectively (*P*<0.05). AA pretreatments at 2-8 μM lowered 14-40% RAGE expression (*P*<0.05); at 1-8 μM it down-regulated p-p38 and p-JNK expression 24-69%, and 22-58%, respectively; dose-dependent manner was apparent in declining p-p38 expression (*P*<0.05).

## 4. Discussion

Glycation is a vital factor in skin aging; potent anti-AGE cosmeceutical compounds have received high interest from pharmaceutical agencies [23]. Our study found asiatic acid pretreatment effectively protecting HaCaT cells, human normal keratinocyte cells, against subsequent AGE-BSA-induced apoptotic, oxidative and/or inflammatory stress. Likewise, this triterpene benefited collagen stability, suppressing AGE-BSA induced activation of RAGE and MAPK pathways in those cells. These novel findings suggest asiatic acid as a potent anti-glycative skin protective agent.

Skin glycation is coupled with oxidative and/or inflammatory response [1,24]. This study revealed AGE-BSA lowering GSH level and stimulating overproduction of ROS, NO and protein carbonyl in HaCaT cells. Both ROS and RNS were apparently involved in glycation associated oxidative stress in this cell line, which not only depleted GSH but also contributed to cell injury and death. We found asiatic acid effectively decreasing formation of ROS, NO, and protein carbonyl. It is highly possible that this triterpene enhanced anti-oxidative defense for those cells, in turn diminishing AGE-BSA to cause oxidative stress and spare GSH. In addition, this triterpene maintained GPX activity and expression, and inhibited NOS activity and iNOS expression. Findings indicate this agent penetrating HaCaT cells and exerting anti-oxidative activities via enzymatic and non-enzymatic actions. On the other hand, we found AGE-BSA causing HaCaT inflammation via greater release of inflammatory cytokines: e.g., IL-1beta, IL-6, TNF-alpha. But asiatic acid pretreatments at 2-8 μM lessened generation of all three. Data reveal this triterpene attenuating glycation associated inflammatory injury. Since both oxidative and inflammatory stress were mitigated, greater viability in asiatic acid-treated HaCaT cells could be explained.

Caspase-3, downstream effector, and caspase-8, upstream initiator in caspase cascade, could act as apoptotic executors in cell death; they are in charge of cell morphological events and nuclear protein cleavage [25]. Alikhani et al. [26] cited AGE-induced apoptosis in fibroblasts as largely dependent on caspase-3 activation. We saw AGE-BSA activating both caspase-3 and -8, enhancing activity and protein expression to facilitate apoptotic process and lead to cell death. Asiatic acid pretreatment at 2-8 μM substantially decreased activity and protein expression of both caspases in HaCaT cells, diminishing apoptotic stress and benefitting survival. Greater viability in treated HaCaT cells could be partially ascribed to this agent decline caspase cascade.

Up-regulated MMPs promote ECM degradation and impede collagen or elastin synthesis in skin, which favors skin aging [9,27]. MMP-1, also called collagenase-1, is chiefly responsible for degradation of collagen I, primary component and structural support of skin dermis [28]. Our study found AGE-BSA raising MMP-1, -8, and -9 expression in HaCaT cells, which in turn enhanced collagen I degradation. Obviously, ECM degradation is involved in glycation-linked skin aging. Data revealed asiatic acid pretreatment at 2-8 μM markedly down-regulating MMP-1, MMP-8 and MMP-9 in this cell line. MMP-1 expression declining explains greater collagen I expression in treated cells. These indicate asiatic acid as beneficial to collagen I and/or ECM stability via suppressing MMPs in skin cells. It is reported that increased MMP-1 and -9 lead to poor wound healing: both impede keratinocyte function [10,29]. Asiatic acid might enhance healing via anti-MMP activity.

In our study, AGE-BSA up-regulated RAGE and MAPK expression in HaCaT cells. Subsequently, the AGE-RAGE interaction and MAPK activation facilitated oxidative and inflammatory reactions, triggering massive production of downstream factors like ROS and inflammatory cytokines. We saw asiatic acid at 2-8 μM down-regulating RAGE expression and consequently reducing available RAGE to react with AGEs. Diminished oxidative and inflammatory stress in asiatic acid treated-HaCaT cells could be partially ascribed to this agent decreasing AGEs-RAGE engagement. Moreover, asiatic acid at 1-8 μM limited p38 and JNK phosphorylation. Obviously, this triterpene attenuates oxidative and inflammatory response via suppressing MAPK pathway: i.e., anti-glycative activity by mediating RAGE and MAPK pathways, while MAPK activation induces MMP expression [30,31]. This triterpene declining in MAPK pathway can partially explain lower MMP expression in treated HaCaT cells. Besides promoting skin aging, glycation impairs healing, especially for diabetics [32,33]. Glycation reportedly aggravates inflammatory cytokine release and delays ECM remodeling in skin wounds [34,35]. We observed asiatic acid’s anti-glycative effects on skin; this triterpene might aid healing by suppressing associated reactions. Asiatic acid, a triterpene, naturally occurs in plant foods. Our data revealed 8 μM asiatic acid alone in HaCaT cells causing no adverse effect; application of this compound for skin protection and/or healing may be safe. Further in vivo study must verify effects and mechanism for skin protection. In sum, pretreatment from asiatic acid protected HaCaT cells against AGE-induced injury. This agent exhibits anti-oxidative, -inflammatory and -apoptotic effects via lowering ROS production, decreasing caspase activity and expression, suppressing RAGE, p38 and/or JNK activation, and down-regulating expression of MMPs. These suggest triterpene as a potent protective agent with antiglycative activity.

## Conflict of Interest

The authors declare no conflicting or competing commercial interests.
